# A three-dimensional bioprinted model to evaluate the effect of stiffness on neuroblastoma cell cluster dynamics and behavior

**DOI:** 10.1038/s41598-020-62986-w

**Published:** 2020-04-14

**Authors:** Ezequiel Monferrer, Susana Martín-Vañó, Aitor Carretero, Andrea García-Lizarribar, Rebeca Burgos-Panadero, Samuel Navarro, Josep Samitier, Rosa Noguera

**Affiliations:** 1 Pathology Department, Medical School, University of Valencia-INCLIVA, Valencia, Spain; 2 CIBERONC, Madrid, Spain; 3 Institute for Bioengineering of Catalonia, Barcelona Institute of Science and Technology (IBEC-BIST), Barcelona, Spain; 4 CIBER-BBN, Madrid, Spain; 5 Department of Electronics and Biomedical Engineering, University of Barcelona, Barcelona, Spain

**Keywords:** Paediatric cancer, Biomaterials - cells

## Abstract

Three-dimensional (3D) bioprinted culture systems allow to accurately control microenvironment components and analyze their effects at cellular and tissue levels. The main objective of this study was to identify, quantify and localize the effects of physical-chemical communication signals between tumor cells and the surrounding biomaterial stiffness over time, defining how aggressiveness increases in SK-N-BE(2) neuroblastoma (NB) cell line. Biomimetic hydrogels with SK-N-BE(2) cells, methacrylated gelatin and increasing concentrations of methacrylated alginate (AlgMA 0%, 1% and 2%) were used. Young’s modulus was used to define the stiffness of bioprinted hydrogels and NB tumors. Stained sections of paraffin-embedded hydrogels were digitally quantified. Human NB and 1% AlgMA hydrogels presented similar Young´s modulus mean, and orthotopic NB mice tumors were equally similar to 0% and 1% AlgMA hydrogels. Porosity increased over time; cell cluster density decreased over time and with stiffness, and cell cluster occupancy generally increased with time and decreased with stiffness. In addition, cell proliferation, mRNA metabolism and antiapoptotic activity advanced over time and with stiffness. Together, this rheological, optical and digital data show the potential of the 3D *in vitro* cell model described herein to infer how intercellular space stiffness patterns drive the clinical behavior associated with NB patients.

## Introduction

The extracellular matrix (ECM) is a three-dimensional (3D) network that forms part of all body tissues. It can be defined as a biophysical filter that provides protection, nutrition and cell innervation, and is involved in immune response, angiogenesis, fibrosis and tissue regeneration. It is also a transport medium for mechanical forces to the basal membrane through integrins that support the tissue tensegrity system, activating cellular genetic and epigenetic mechanisms^1^. ECM alterations result in loss of functions such as cell denervation^2^, loss of regeneration and wound-healing capacity^3^, loss of the substrate that provides a correct immune response to infectious^4^, tumoral^5^ and toxic agents^6^ and alteration of mechanical transmission (mechanotransduction changes)^7^. 2D cell culture has been one of the most commonly used *in vitro* models for biomedical research, due to its ease of use and low cost; however, it is less effective in reflecting the effect of the ECM and potential cellular microenvironment interactions, being unable to capture the interaction between 3D architecture of cells and ECM^8^. 3D cell culture has been used to show that ECM rigidity may enhance cell motility by modifying their morphological properties to an aggressive phenotype^9–11^. Furthermore, 3D cell culture has already been used to study the impact of the ECM on cancers such as breast cancer^12^, sarcoma^13^ and pancreatic cancer^14^. From this approach, tumors can be studied as functional tissues, connected to and dependent on the microenvironment. Regarding model fabrication, 3D bioprinting technology has certain advantages over casted 3D gels, with the first technology permitting direct cell incorporation and homogeneous cell distribution in the model, preparation at room temperature and design of precisely defined mesh structures to facilitate nutrient flow to the cells^15^. Thus 3D bioprinting technology can contribute towards standardizing medical devices^16^. These 3D microenvironments mimicking human tumors can be analyzed using several parameters such as Young’s modulus, a parameter that characterizes the behavior of elastic material, used to define the stiffness of bioprinted hydrogels and human tumors^17,18^ and tumor cell proliferation biomarkers, that can be easily studied by immunohistochemical (IHC) analysis of the Ki67 marker^19–22^, as well as via the following: (i) polypyrimidine tract binding protein 1 (PTBP1) staining, which is associated with pre-mRNAs in the nucleus and influences pre-mRNA processing and some aspects of mRNA metabolism and transport^23–26^. High PTBP1 expression has been associated with aggressive behavior in several types of cancer, especially breast cancer, glioma and ovarian tumors^27,28^; (ii) the mitosis-karyorrhexis index (MKI), defined as the cellular density sum of mitotic and karyorrhectic cells in a tumor. A high MKI is an indicator of poor prognosis in cancers such as neuroblastoma (NB)^29–31^; and finally, (iii) Bax and Bcl2 markers, used to characterize cellular signals of apoptosis and antiapoptosis activity, respectively^32–35^.

NB is among the most common solid cancers in childhood, with a wide variety of presentations and highly variable prognosis, depending largely on anatomical location in the sympathetic nervous system where the primary tumor develops, and metastatic status^36^. Malignant neuroblastic cells are highly sensitive to the biomechanical properties of their microenvironment^9,37^ and this was verified in our studies, where we observed that the composition of the ECM can define an ultra-high-risk subset within the high-risk group of neuroblastoma patients (HR-NB)^38^, and that a stiff ECM can be generated and associated with aggressive neuroblastic tumors^39–41^. Paradoxically, the ECM is not taken into account in standard cancer management practice today, despite evidence pointing to a key role for the ECM during tumor progression and therapy resistance^42^. The use of 3D cell culture with different hydrogel stiffness could help us characterize the effects of ECM stiffness on malignant neuroblastic cell behavior, as well as providing a way to simulate and better understand the biomechanical properties found in HR-NB tumor tissues. In this study we used morphometric digital analysis to evaluate the different effects of ECM stiffness on NB cells over time, using a 3D scaffold-based *in vitro* cell culture platform, demonstrating its value in molecular mechanotherapy evaluation.

## Methods

### 2D and 3D culture of SK-N-BE(2) cells

SK-N-BE(2) cells were acquired from American Type Culture Collection (ATCC, Manassas, VA, USA) and expanded in a growth medium based on Iscove’s Modified Dulbecco’s Medium (IMDM, Gibco, Thermofisher), supplemented with 10% fetal bovine serum (Thermofisher), 1% Insulin-Transferrin-Selenium G Supplement (Thermofisher), Plasmocin (0.2%) treatment ant-mpt (1/10) (InvivoGen) and 1% penicillin/streptomycin (Thermofisher) at 37 °C and 5% CO_2_ atmosphere. 2D cell cultures were grown in 8-well Cell Culture Slides (SPL Life Sciences) until they reached confluence before immunocytochemistry (ICC) analysis.

To create the bioinks, cells were cultured and trypsinized. The resulting pellet was resuspended with the prepolymer solution at 37 °C to a 2.5 × 10^6^ cell density. The bioink was loaded in a bioprinting syringe and gelified at −20 °C for 3 minutes before printing.

### Synthesis of hydrogels

Methacrylated gelatin (GelMA), a photocrosslinkable hydrogel derived from natural gelatin, emulates the ECM for various cells types in combination with non-biodegradable materials such as alginate. We used the synthesis process as previously described^43^. Briefly, Gelatin (Sigma-Aldrich, USA) was modified to a 40% degree of methacrylation. First the Gelatin was dissolved in PBS 10 mM at a concentration of 10% (w/v), and mixed drop by drop with methacrylic anhydride (Sigma-Aldrich, USA)^44^. One hour later, the reaction was stopped by adding an excess of PBS 10 mM and was dialysed against Milli Q water with 6–8 kDa MWCO membranes (Spectra/por, Spectrumlabs, USA). Sodium alginate (Sigma-Aldrich) was methacrylated (AlgMA) at a maximum degree of methacrylation as previously described^45^. The methacrylation reaction was performed by mixing a solution of 1% (w/v) of the polymer in 50 mM MES buffer at pH 6.5 with 20 mM EDC, 10 mM N-hydroxysuccinimide and 10 mM 2 aminoethylmethacrylate (Sigma-Aldrich). The reaction was stopped after 24 h with the addition of acetone (Panreac, Spain) and filtered using a vacuum flask. The precipitate was dissolved in PBS 10 mM and dyalised against Milli Q water with 3.5 kDa MWCO membranes (Thermofisher, USA). Finally, the solutions of methacrylated polymers were lyophilized and stored at −20 °C. The polymer precursors (GelMA and AlgMA) were mixed at different concentrations and diluted in growth medium containing the photoinitiator, lithium Phenyl (2,4,6 trimethylbenzoyl) phosphinate (LAP) (TCI EUROPE N.V., Belgium), in a concentration of 0.05% w/v^43^.

These prepolymer solutions were placed at 65 °C for 1 h to obtain homogeneous solutions. Prepolymer solutions were prepared to obtain final concentrations of 5% w/v GelMA and 0%, 1% and 2% w/v AlgMA according to the desired initial stiffness level (the higher the alginate percentage, the stiffer the hydrogel). Prepolymer compositions were mixed with cells to generate the gelified bioinks. All hydrogels were fabricated using a 3D bioprinter (3DDiscovery BioSafety, regenHU, Switzerland; 365 nm, 3 W cm^−2^) polymerized with UV light source as previously reported^43^. Briefly, cell-laden gelified bioinks were printed applying an air pressure extrusion system, using a 150 μm nozzle. The bioprinter generated 0.4 mm spaced bioink rows to make 5 × 5 mm layers. Successive layers were photocrosslinked by 5 s exposure to UV light at 3 W cm^−2^ and printed perpendicularly to generate a 1 mm-high 5-layer network. Next, hydrogels were immersed in growth media to remove unreacted reagents, and then cultured for 2 and 4 weeks before analysis.

### Scanning electron microscope

The matrix pore of the three hydrogels was studied with scanning electron microscope (SEM) images. 5% GelMA with 0%, 1% and 2% AlgMA hydrogels were bioprinted as explained previously and incubated in PBS1X for 24 h until they reached the equilibrium swelling state. Hydrogels were dehydrated using a series of ethanol gradient with distilled water: 30%, 50%, 70%, 90%, 96% and 100% for 10 minutes. The critical dry point (K850, Quorum technologies, UK) was obtained by replacing the ethanol by liquid CO_2_. The CO_2_ was slowly evaporated under 85.06 atm pressure and 35 °C to completely dry the hydrogels. Next, hydrogels were gold-spattered and images were taken with ultrahigh resolution SEM (Nova NanoSEM 230, FEI Company, The Netherlands). Pore size distribution was analyzed after processing the images with ImageJ open software.

### Mechanical compression assays

**Figure 1:**
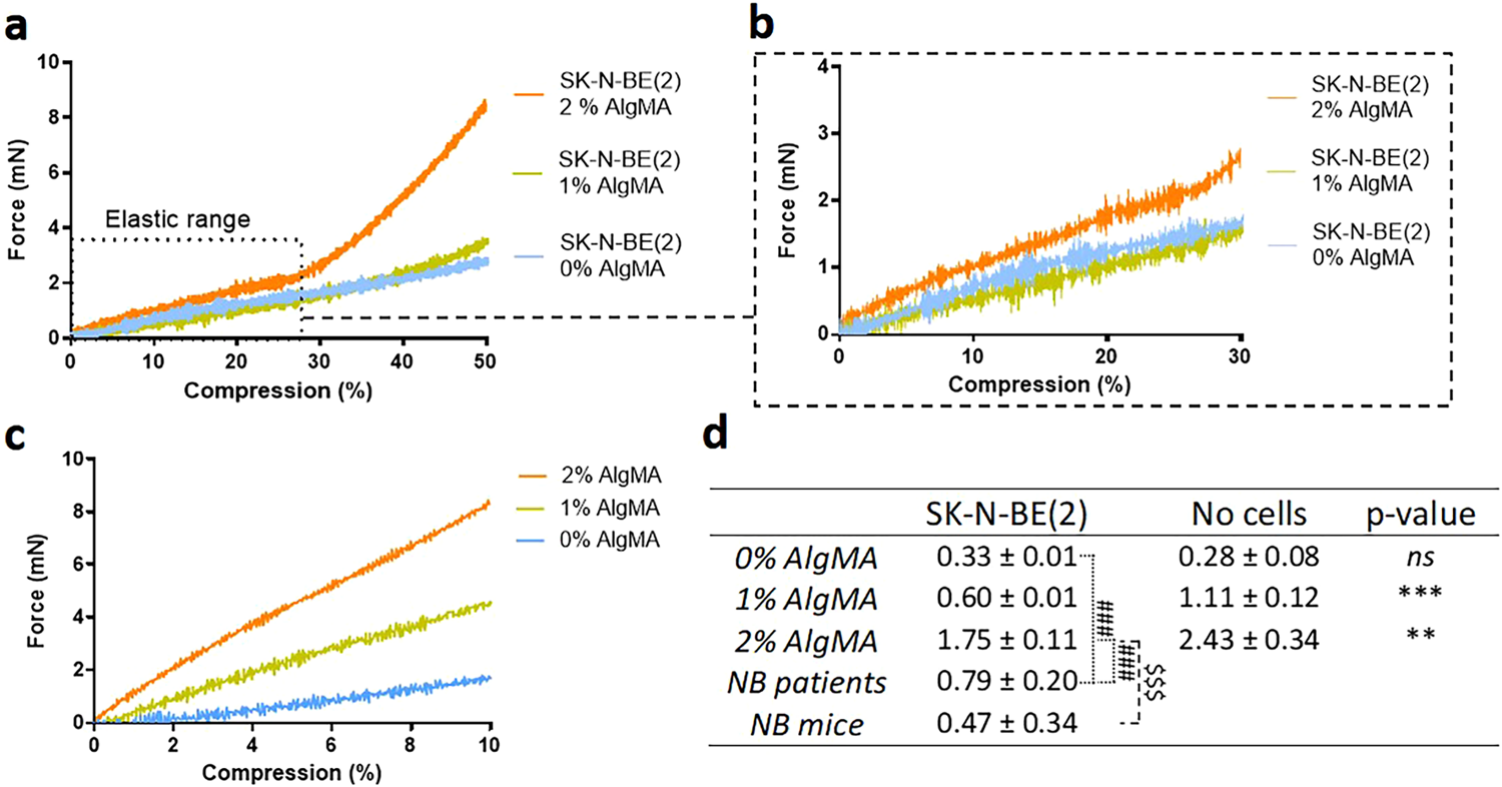
(**a**–**c**) Strain-stress curves obtained by testXpert after the compressive test. (**a**) Representation of the average curves of the 3 hydrogel types: 0%, 1% and 2% AlgMA with SK-N-BE(2). Young’s Modulus was obtained from the linear range of the elastic region. (**b**) Zoom view of the linear range of the elastic region of SK-N-BE(2) laden hydrogels. (**c**) Zoom view of the linear range of the elastic region in hydrogels without cells. (**d**) Characterization of the mechanical properties (Young’s modulus) of the composite hydrogels, cohort of HR-NB and orthotopic NB mice tumors. Comparative Young´s modulus of 5% gelatin methacrylated with 0%, 1% and 2% methacrylated alginate in absence and presence of incubated SK-N-BE(2) cells. Measurements of each hydrogel were performed in quintuplicate. Values are plotted as mean and standard deviation. Statistical analysis using *t-student* test: ^ns^*p*-value > 0.05, ^**^p-value < 0.01, ***p-value < 0.001, comparing SK-NB-E(2) and no cell-hydrogels, ^###^*p*-value < 0.001, comparing NB patients vs SK-NB-E(2) hydrogels and ^$$$^*p*-value < 0.001, comparing orthotopic NB mice tumors vs SK-NB-E(2) hydrogels.

Mechanical properties of hydrogels were determined by uniaxial compression assays using a Zwick Z0.5 TN instrument (Zwick-Roell, Germany) with a 5 N load cell, as previously described^43^. Hydrogels were bioprinted as explained above and incubated at 37 °C to reach the equilibrium swelling state before measurement. The hydrogels were compressed up to 50% of deformation, with a 0.1 mN preload force and 20%/min strain rate. As there were no significant variations between samples measured at 37 °C (Supplementary Fig. 1a), all measurements were performed at room temperature submerged in a medium drop. Young’s modulus was calculated from the slope of the elastic region of stress-strain curves^46^ using the testXpert software (Zwick-Roell) (Fig. 1a–c). Measurements of each hydrogel were performed in quintuplicate. Compressive modulus was obtained from four different material groups: i) hydrogels 5% GelMA with 0% AlgMA, 1% AlgMA and 2% AlgMA in absence of cells incubated for 48 hours and ii) with SK-N-BE(2) cells incubated for 24 days, iii) 6 HR-NB human tumors and iv) 6 orthotopic SK-N-BE(2) and SH-SY5Y cells from xenograft-derived tumors. Both HR-NB cohort and orthotopic NB mice tumors were used as comparison groups; 3 out of 6 tumors in each group showed *MYCN* amplification (MNA)^47^. The statistical analysis was performed using t-student test.

### Paraffin embedding, histochemical analysis and optical microscopy quantification

Hydrogels were collected and placed in Tissue-Tek Paraform biopsy cassettes (Sakura Finetek, USA), fixed in 4% formaldehyde and automatically embedded in paraffin (Leica EG1150H; Leica Microsystems; Wetzlas, Germany). Paraffin-embedded samples were cut into 3 μm sections. Hematoxylin-eosin staining (HE) was performed for morphology and MKI studies. MKI was determined by an expert pathologist in accordance with the Shimada classification^48^: low MKI (<100 MK cells/5000 cells or <2%); intermediate MKI (100–200 MK cells/5000 cells or 2–4%), or high MKI (>200 MK cells/5000 cells or >4%). Automated IHC and ICC stains (Autostainer Link 48; Dako, Glostrup, Denmark) using anti-Ki67 (prediluted), anti-PTBP1 (dil. 1/400), anti-Bax (dil. 1/50) and anti-Bcl2 (prediluted) antibodies, all from Dako (Agilent Technologies, USA), were quantified by optical microscopy. For IHC and ICC markers, cells stained in blue indicated negative cells while brown staining was considered a positive result. Samples were examined and interpreted using the following criteria: - Negative (<1% positive cells); + Low positive (1–20% positive cells); ++ Intermediate positive (20–50% positive cells); +++ High positive (>50% positive cells).

### Image analysis

HE, Ki67 and PTBP1 stained sections were digitalized with the whole-slide Pannoramic MIDI scanner (3DHISTECH Ltd., Budapest, Hungary) at 20x magnification. Detected artefacts and folded and/or broken regions were considered uninformative and were excluded. HistoQuant module was applied in HE-stained sections to obtain the solid area of hydrogels, defining solid area as the regions of the sample with cellular clusters and/or biopolymers. The number of cellular clusters, their respective areas and total hydrogel area for each condition were obtained by segmentation with Pannoramic Viewer (PV) software (3DHISTECH) (Supplementary Fig. 2). Cell nucleus size and shape were also obtained with the HistoQuant module of the PV software. Ki67 and PTBP1 were also determined using PV software, and their expression-related parameters (number of positive cells) were analyzed automatically applying the NuclearQuant module.

### Data treatment

All data from the 3D models was analyzed by comparing i) different timeframes with the same stiffness and ii) different stiffness within each culturing timeframe. 3D data was also compared with the 2D data. In order to define bioprinted hydrogel characteristics and cell behavior, three informative parameters were established: (i) hydrogel porosity, (ii) cell cluster density and (iii) cell cluster occupancy. All these variables were calculated from HE-stained samples. We defined hydrogel porosity as the amount of holes in the hydrogel and was calculated as hollow area/total area percentage, the hollow area representing the total hydrogel area minus hydrogel solid area. Cell clusters were grouped by size and cell number into small (<400 µm^2^; <10 cells), medium (400–2,000 µm^2^; 10–50 cells) or large (>2,000 µm^2^; >50 cells) and cell cluster parameters were calculated for each group. Cell cluster density is a quantitative way to indicate cell cluster presence, calculated as the number of cell clusters/hydrogel solid area (mm^2^). Moreover, percentages of different cell cluster sizes were calculated for each stiffness condition under study as an additional variable of cell cluster presence. Cell cluster occupancy quantifies the hydrogel solid area occupied by cell clusters and was calculated as the percentage of cluster area/hydrogel solid area (mm^2^) for each condition. Regarding Ki67 and PTBP1 expression-related analysis, positive cell percentages were compared for each condition under study. We calculated the overall percentage of Ki67 and PTBP1 positive cells in each sample and their percentages in each cellular cluster size group. Furthermore, H-score was automatically calculated with Pannoramic Viewer in Ki67 and PTBP1 stained samples according to the percentage of positive cells and their staining intensity. This H-score has a value within the range 0–300, where 0 equals the minimum positivity value and staining intensity and 300 the maximum.

A schematic diagram of the complete methodology used for this study is shown in Supplementary Fig. 3.

## Results

### Characterization of bioprinted hydrogels

#### Mechanical properties

For hydrogels printed in the absence of cells and with 48 h culture, Young’s modulus was 0.28 ± 0.08 kPa for 0% AlgMA, 1.11 ± 0.12 kPa for 1% AlgMA and 2.43 ± 0.34 kPa for 2% AlgMA. After 24 days of cell incubation, results were 0.33 ± 0.01 kPa for 0% AlgMA, 0.60 ± 0.005 kPa for 1% AlgMA and 1.75 ± 0.11 kPa for 2% AlgMA. Differences in elastic modulus between composite hydrogels with and without cells were statistically significant (p < 0.05) in 1% AlgMA and 2% AlgMA (Fig. 1). We obtained a Young´s modulus mean of 0.79 ± 0.20 kPa for HR-NB tumors, in a range between 0.58 to 1.1 kPa. Comparing these results with those obtained in bioprinted hydrogels, we observed that the mean of Young´s modulus in human NB was similar to that of the bioprinted hydrogels composed of 1% of AlgMA (Fig. 1). Regarding NB mice tumors, we obtained a Young´s modulus mean of 0.47 ± 0.34 kPa in a range between 0.06 to 1.05 kPa, similar to the one of bioprinted hydrogels composed of 0% and 1% of AlgMA (Fig. 1). Therefore, we found that cell presence in the bioink decreased Young’s modulus and allowed 0% AlgMA and 1% AlgMA hydrogels to better imitate the mechanical properties of human HR-NB tumors and mice tumors. Supplementary Fig. 1b shows a bar chart of the mechanical properties.

#### Matrix pore

**Figure 2:**
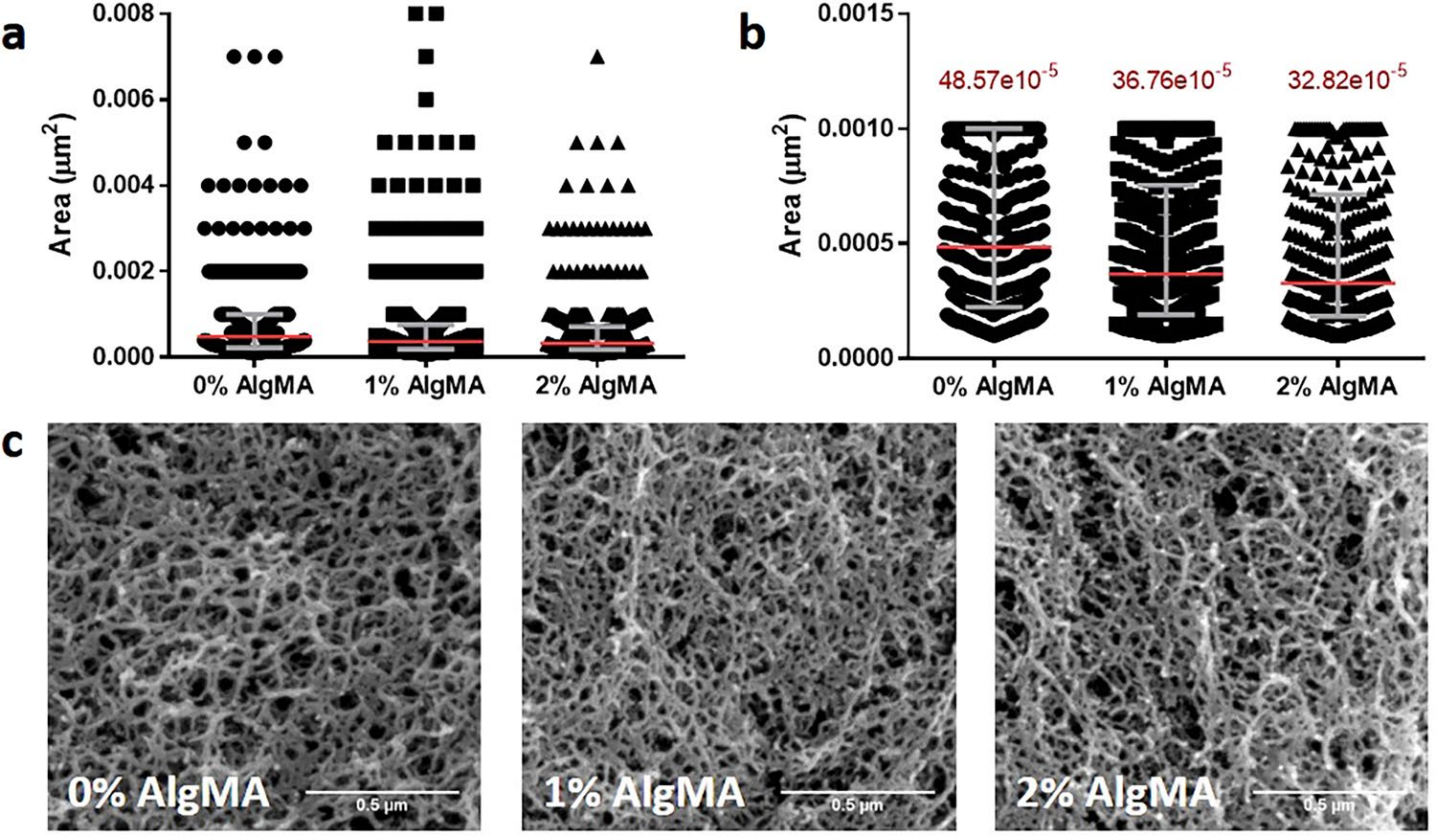
(**a**) Pore area dot plot of 0%, 1% and 2% AlgMA hydrogels and (**b**) zoom in of the interquartile region. The interquartile region is shown in grey and the median in red. (**c**) SEM images of the three hydrogel combinations.

The fiber mesh of the 5% GelMA with 0%, 1% and 2% AlgMA biomaterials were studied using SEM images. The polymer fibers presented an interconnected mesh with a wide pore size range, so we studied the distribution of the pore area data instead of the average pore size of each composite. All the samples contained pores from the smallest to the largest areas and all showed right-skewed data distribution (Fig. 2a). However, in 0% AlgMA the sample median was higher (Fig. 2b), meaning that the data was biased towards higher area values. 1% AlgMA also showed a higher median than 2% AlgMA, but the difference was less notable. The interquartile range of 1% AlgMA and 2% AlgMA was narrower than 0% AlgMA, suggesting that most data was condensed in the small area values. Hence, the frequency of large pores decreased when the alginate concentration was increased. This was also observed in the SEM images, where 0% AlgMA showed higher number of large pores (Fig. 2c).

#### Porosity

**Figure 3:**
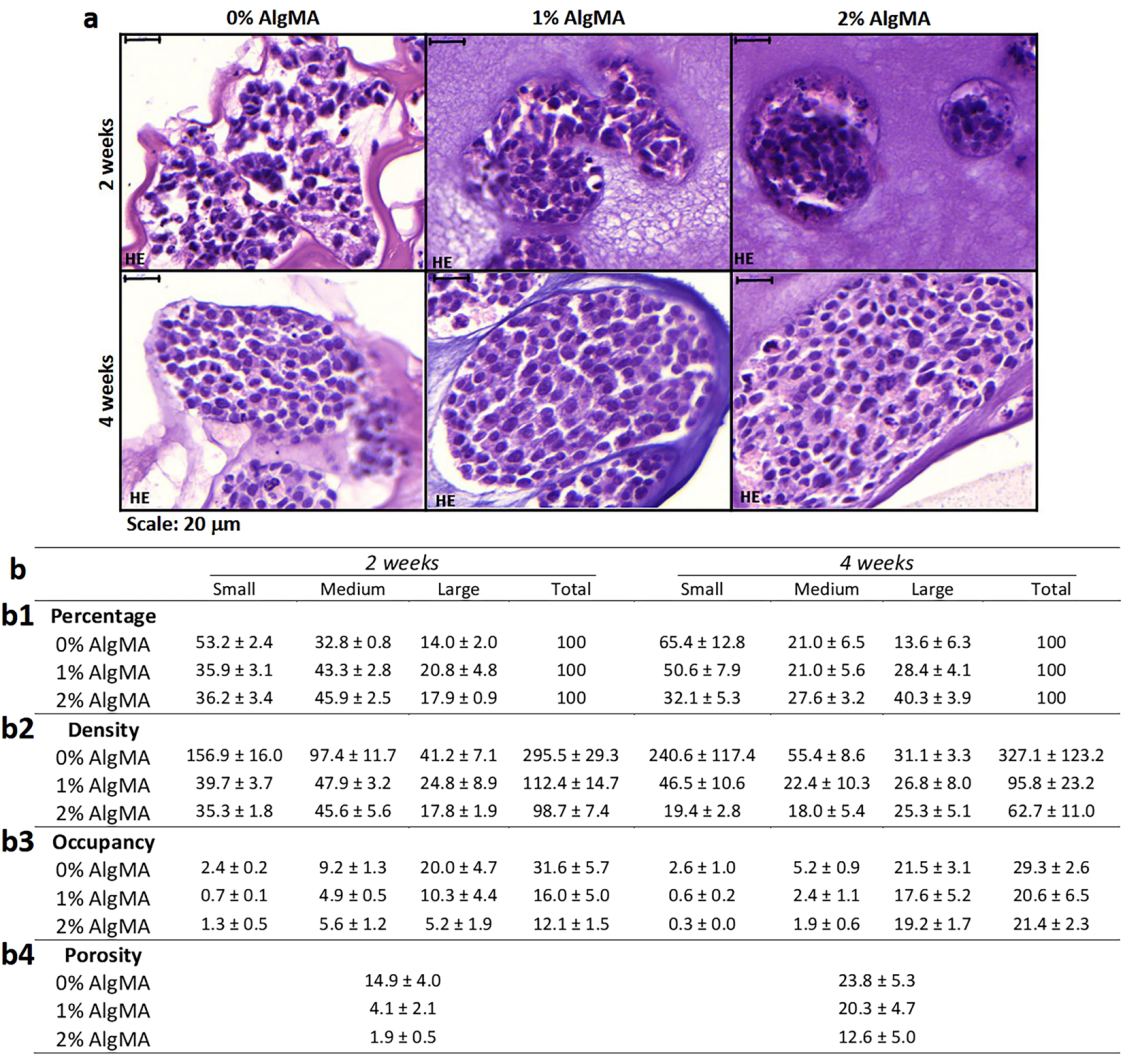
Characterization of bioprinted hydrogels. (**a**) Hydrogels hematoxylin-eosin stains (80×) studying the morphology of the models. (**b**) Percentage of clusters (**b1**), cluster density (**b2**), percentage of occupancy **(b3**) for each cluster size (small: <400; medium: 400–2,000; large: >2,000 μm^2^) and (**b4**) porosity of bioprinted hydrogels. Values are plotted as mean and standard error of the mean.

Differences in porosity observed by optical microscopy were confirmed and quantified by image analysis. The results showed that porosity increased with time, from 14.9% ± 4.0, 4.1% ± 2.1 and 1.9% ± 0.5 at 2 weeks to 23.8% ± 5.3, 20.3% ± 4.7 and 12.6% ± 5.0 at 4 weeks, ordered from low to high initial stiffness condition (Fig. 3b-b4). This indicates that over the same timeframe, as alginate in the initial hydrogel composition increased, the hydrogel became more compact and therefore less porous. Differences in porosity between stiffness conditions were more evident at 2 weeks, which was closer to the time when the hydrogels were created and they had therefore undergone fewer changes. Highest porosity was achieved after 4 weeks, with similar porosities (22.0% ± 3.3) at 0% and 1% AlgMA (Fig. 3b-b4). The results also showed that 0% AlgMA was the stiffness condition with least variation in porosity over time (8.9% increase) and 1% and 2% AlgMA had 16.2% and 10.7% increase respectively (Fig. 3b-b4). Supplementary Fig. 4d shows bar charts of porosity.

### Cluster dynamics over time and by hydrogel stiffness

#### Cluster density pattern

We observed with optical microscopy that in 0% AlgMA hydrogel clusters were small, irregular and occupied a large surface of the model. In contrast, 1% and 2% AlgMA hydrogels showed large, round and compact clusters occupying a smaller surface of the model (Fig. 3a).

These parameters were validated and mathematically quantified by digital analysis. Comparing timeframes within the same stiffness showed that hydrogels with 0% AlgMA represented the highest percentage of small clusters (53.2% ± 2.4 and 65.4% ± 12.8) and the smallest percentage of large clusters (14.0% ± 2.0 and 13.6% ± 6.3), at both 2 and 4 weeks culturing, respectively (Fig. 3b-b1). When considering cluster density variation in 0% AlgMA, we observed a high variability on the total number of clusters over time (from 295.5 ± 29.3 to 327.1 ± 123.2 clusters/mm^2^) and a decrease in medium and large cluster sizes (from 97.4 ± 11.7 to 55.4 ± 8.6 and 41.2 ± 7.1 to 31.1 ± 3.3 clusters/mm^2^) (Fig. 3b-b2). At 1% AlgMA, all cluster size percentages (33.3% ± 13.5) (Fig. 3b-b1) but also total and size-related cluster density (104.1 ± 31.4 and 34.7 ± 15.8 clusters/mm^2^, respectively) were maintained over the culturing period, with minimal variation within the same size over time (i.e.: 24.8 ± 8.9–26.8 ± 8.0 clusters/mm^2^ in large clusters) except medium sized clusters, displaying the highest variations (22.3% and 25.5 clusters/mm^2^ variation over time) (Fig. 3b-b2). Finally, at 2% AlgMA large cluster percentages increased noticeably over time compared to medium and small (17.9% ± 0.9 to 40.3% ± 3.9) (Fig. 3b-b1). We also observed that total cluster density decreased over the timeframe (98.7 ± 7.4 to 62.7 ± 11.0 clusters/mm^2^), driven by a decrease in small and medium clusters (from 35.3 ± 1.8 to 19.4 ± 2.8 and 45.6 ± 5.6 to 18.0 ± 5.4 clusters/mm^2^, respectively) and increased in large ones (from 17.8 ± 1.9 to 25.3 ± 5.1 clusters/mm^2^) (Fig. 3b-b2).

Comparing different stiffness at the same time point showed that at 2 weeks, larger clusters were a minority in all stiffness conditions (14.0% ± 2.0 in 0% AlgMA, 20.8% ± 4.8 in 1% AlgMA and 17.9% ± 0.9 in 2% AlgMA) (Fig. 3b-b1). Furthermore, we observed that the stiffer the hydrogel, the lower the density in all clusters (i.e.: 156.9 ± 16.0–39.7 ± 3.7–35.3 ± 1.8 clusters/mm^2^ in small clusters) (Fig. 3b-b2). At 4 weeks, each cluster size group behaved differently as stiffness increased: while the proportion of small clusters was reduced (65.4% ± 12.8–50.6% ± 7.9–32.1% ± 5.3), the medium clusters remained constant (21.0% ± 6.5–21.0% ± 5.6–27.6% ± 3.2) and large clusters increased (13.6% ± 6.3–28.4% ± 4.1–40.3% ± 3.9) (Fig. 3b-b1). Furthermore, at 4 weeks cluster density also decreased with stiffness. However, it remained more stable as the clusters increased in size (240.6 ± 117.4–46.5 ± 10.6–19.4 ± 2.8 clusters/mm^2^ in small clusters, 55.4 ± 8.6 – 22.4 ± 10.3–18.0 ± 5.4 clusters/mm^2^ in medium clusters and 31.1 ± 3.1–26.8 ± 8.0–25.3 ± 5.1 clusters/mm^2^ in large ones) (Fig. 3b-b2). Supplementary Fig. 4a,b shows bar charts of cluster density pattern.

#### Percentage occupancy

Firstly, we studied the effect of time on cluster occupancy within the same stiffness. At 0% and 1% AlgMA it was observed that large clusters had the highest occupancy at all-time points (20.0% ± 4.7 and 10.3% ± 4.4 at 2 weeks and 21.5% ± 3.1 and 17.6% ± 5.2 at 4 weeks) (Fig. 3b-b3). In the case of 2% AlgMA hydrogels, medium and large clusters occupied a similar area at 2 weeks (5.6% ± 1.2 and 5.2% ± 1.9 respectively), slightly dominated by medium clusters, but the area increased at 4 weeks, dominated by large clusters (19.2% ± 1.7) (Fig. 3b-b3). Additionally, we could observe that time (2 vs 4 weeks) did not affect the total occupancy in 0% AlgMA (31.6% ± 5.7 vs 29.3% ± 2.6) and 1% AlgMA (16.0% ± 5.0 vs 20.6% ± 6.5), but a positive one in 2% AlgMA (12.1% ± 1.5 vs 21.4% ± 2.3).

We also studied the percentage of occupancy and the effect of stiffness within the same culturing period. At 2 weeks we found that generally the stiffer the hydrogel was, the lower percentage of space it occupied (i.e.: 20.0% ± 4.7–10.3% ± 4.4–5.2% ± 1.9 in large clusters) (Fig. 3b-b3). At 4 weeks slightly lower occupancy was observed in the 1% and 2% AlgMA hydrogels, although in general the percentages were similar (i.e.: 21.5% ± 3.1–17.6% ± 5.2–19.2% ± 1.7 in large clusters, with increasing stiffness) (Fig. 3b-b3). Supplementary Fig. 4c shows bar charts of percentage occupancy.

### Clustered cell behavior across timeframes and by hydrogel stiffness

#### Cell nucleus characterization

Changes in nucleus size and shape were evaluated by digitally quantifying their area and shape factor, revealing minimal variations in either parameter when compared with time and stiffness. Nucleus areas in 0% AlgMA hydrogels varied from 14.04 ± 11.20 μm^2^ to 13.61 ± 11.20 μm^2^, in 1% AlgMA from 17.96 ± 49.62 μm^2^ to 15.92 ± 18.26 μm^2^ and in 2% AlgMA from 13.02 ± 8.45 μm^2^ to 14.88 ± 48.35 μm^2^ at 2 and 4 weeks respectively. The shape factor increase indicated that nuclei tended to become more irregular with time and stiffness, but changes were minimal. We found that shape factor in 0% AlgMA hydrogels varied from 0.50 ± 0.20 to 0.63 ± 0.21, in 1% AlgMA from 0.55 ± 0.21 to 0.61 ± 0.23 and in 2% AlgMA from 0.62 ± 0.20 to 0.71 ± 0.19 when comparing values at 2 and 4 weeks respectively.

#### Cell proliferation (Ki67)

**Figure 4:**
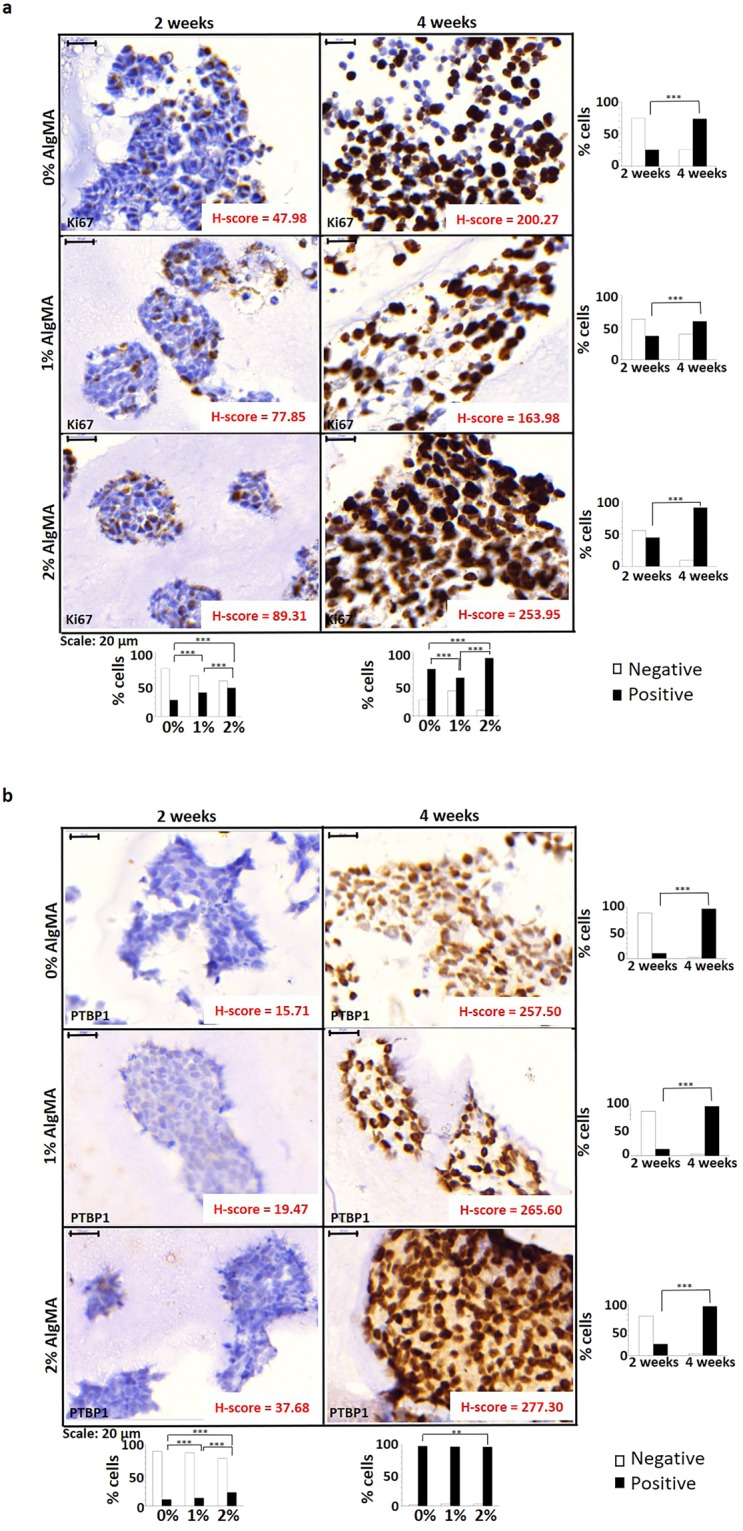
Study of cell proliferation. Immunohistochemistry images (80×), percentage of negative and positive cells and H-score for (**a**) Ki67 and (**b**) PTBP1. Results expressed as percentage of cells represent total number of cells. White bars: negative cells; Black bars: positive cells. Statistical analysis using *Χ*^2^ test: **p-value < 0.01, ***p-value < 0.001.

2D cell cultures presented a high proliferation rate (80% positive cells). When cells cultured in hydrogels were analyzed, in all the stiffness considered Ki67 positive cells increased over time by optical microscopy, with a predominantly peripheral cluster distribution. Digital analysis verified and quantified the increase in positive cells and H-score when culturing times and stiffness increased. From 2 to 4 weeks, positive cell percentages increased threefold at 0% AlgMA (from 25.5% to 73.8%; H-score from 47.98 to 200.27), approximately twofold at 1% AlgMA (from 36.9% to 59.8%; H-score from 77.85 to 163.98) and twofold at 2% AlgMA (from 44.5% to 90.7%; H-score from 89.31 to 253.95) (Fig. 4a). The percentage of Ki67 positive and negative cells was also studied across different cluster sizes. No direct effect of the cluster area on percentages of positive and negative cells was observed in any of the conditions studied (Supplementary Fig. 5a).

#### Polypyrimidine tract binding protein 1 (PTBP1)

As previously described for Ki67, SK-N-BE(2) cells presented a high mRNA metabolism (90% positive cells) in 2D culture. Furthermore, there was a significant increase in positive hydrogel cultured cells from 2 to 4 weeks culturing in all stiffness conditions (from 15.2 ± 6.3% to 97.2 ± 0.7%). At 2 weeks, the increase in stiffness also increased the number of positive cells significantly, from 10.3% in 0% AlgMA to 22.3% in 2% AlgMA, increasing the H-score from 15.71 to 37.68 as well. However, at 4 weeks the percentage of positive cells were similar in all stiffness conditions (97.2 ± 0.7%), with minimal increase in the H-score related with high stiffness (from 257.50 to 277.30) (Fig. 4b). In addition, different cluster sizes did not affect the percentage of stained cells in the 0% AlgMA hydrogels either at 2 or at 4 weeks. By contrast, at 2 weeks the hydrogels with 1% and 2% AlgMA evinced a significant decrease in positive cells as the size of the clusters increased (33.8%-21.3%-3.3% and 48.2%-39.6%-9.1%, respectively); this behavior was not maintained at 4 weeks (Supplementary Fig. 5b).

#### Mitosis-Karyorrehexis index

A high MKI score was determined by optical microscopy (MK cells>4%) and showed that the prevalence of cells in karyorrhexis was much higher than the number of mitotic cells in 2D culture (24% vs 2%) and in all hydrogel samples (28.8 ± 14.3% vs 1.4 ± 0.9%), with the highest MKI in 2% AlgMA at 4 weeks of culture (48.6% MK cells). Results are shown in Table 1.

**Table 1:** MKI results for bioprinted hydrogels. Percentage MK cells > 4% represents high MKI. w: weeks; H: high.

Hydrogel conditions	% Karyorrhexis Cells	% Mitosis Cells	% MK Cells	MKI Classification
0% 2 w	19.6	2.4	22	**H**
1% 2 w	45	2.6	47.6	**H**
2% 2 w	28.2	0.6	28.8	**H**
0% 4 w	20	1.6	21.6	**H**
1% 4 w	12.8	0.2	13	**H**
2% 4 w	47.4	1.2	48.6	**H**

w: weeks; H: high.

#### Apoptosis and antiapoptosis markers

**Figure 5:**
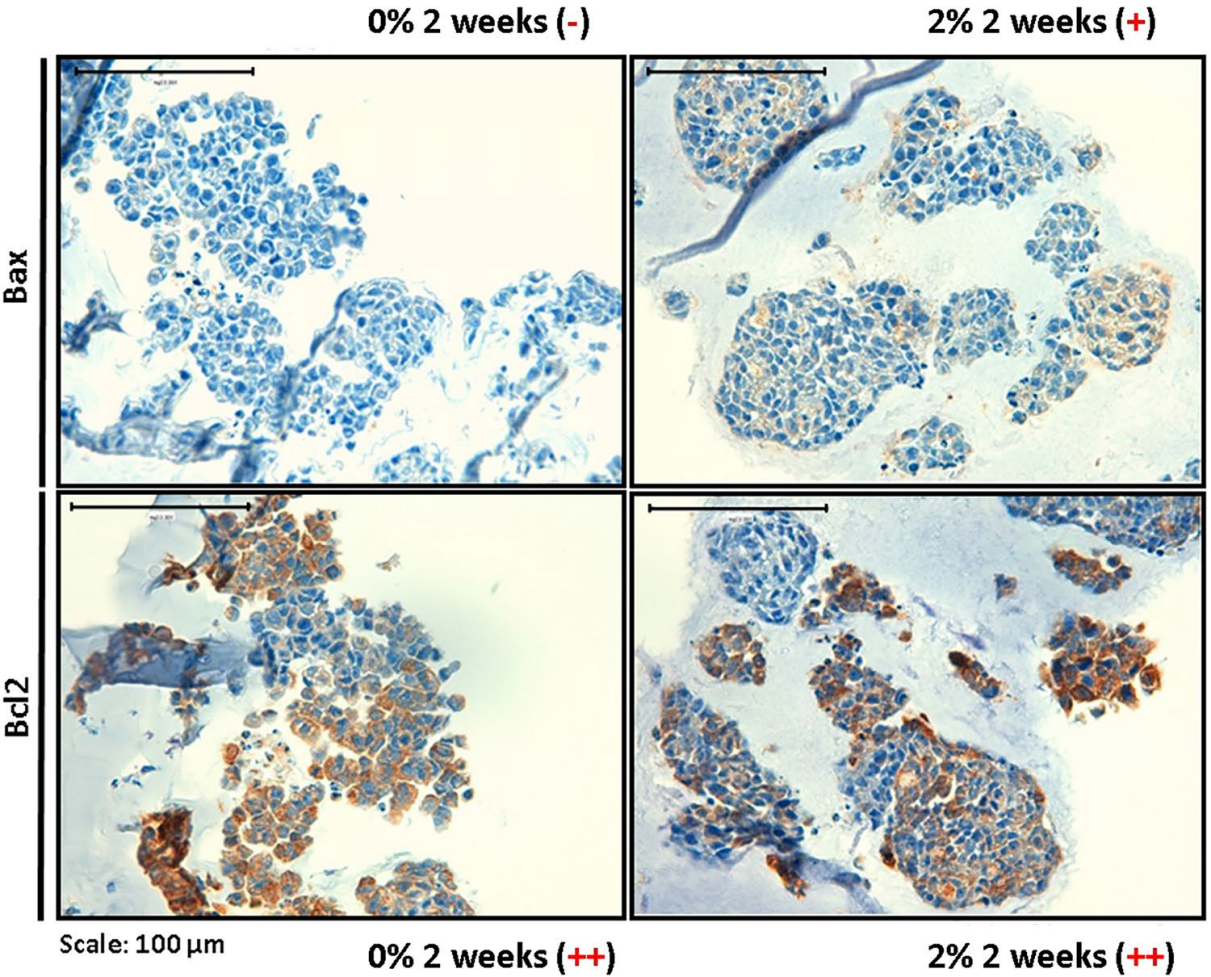
Study of apoptosis and antiapoptosis. Immunohistochemistry images (40×) of Bax and Bcl2 markers. Optical microscopy analysis: − Negative (<1% positive cells); + Low positive (1–20% positive cells); ++ Intermediate positive (20–50% positive cells).

2D cell cultures presented high Bcl2 expression (95% of positive cells) and intermediated Bax expression (40% of positive cells). Optical microscopy in hydrogel samples also revealed a greater number of antiapoptosis markers (Bcl2) than apoptosis markers (Bax), increasing with stiffness (Fig. 5). Nevertheless, the amount of positive cells in 3D culture was much lower than in 2D culture, while Bcl2 positive cells remained between 20% and 50%, 100% of Bax negative cells were present in 0% AlgMA and Bax positive cells varied between 1% and 20% in 2% AlgMA.

## Discussion

The detailed mechanisms by which a physical stimulus is converted into a biochemical response in cancer are not well understood, consequently, molecular events occurring in the tumor microenvironment during pathogenesis and tumor progression, such as cell-cell/ECM interactions or fluid shear forces, are a current focus of research^49^. Standard *in vivo* and *in vitro* models are traditionally used to elucidate the mechanisms involved in pathobiology and evaluate chemotherapeutics. However, the individual contribution of interconnected physical and biochemical parameters cannot easily be assessed in *in vivo* models^50^ and they also fail to predict the clinical efficacy of new drug candidates. The challenge in treating HR-NB is gaining insight into structural, molecular and microenvironmental changes that can be applied to halt tumor metastasis and resistance to multiple chemotherapeutic drugs. 3D scaffold-based NB cell models have only recently been used in studies^51,52^ yet provide valuable platforms for analyzing the biological signs underlying disease progression. In the present study we successfully characterized SK-N-BE(2) neuroblastic cell behavior in 3D culture, using bioprinted hydrogels composed from GelMA and different percentages of AlgMA to represent various ECM initial stiffness conditions. SK-N-BE(2) cells present MNA, a well-known marker of aggressiveness in NB. SK-N-BE(2) cells maintained MNA in our 3D culture conditions, and therefore this cell line is suitable to study ECM mechanical properties in relation to the MNA NB tumor microenvironment in a patient cohort. Furthermore, as the cultured cells can be removed from the scaffolds by trypsin-mediated hydrogel degradation, the bioprinted 3D models presented herein permit varied study with isolated cell clusters.

This study confirms the capacity of GelMA and different percentages of AlgMA scaffolds to support NB cell line growth, as previously described in the collagen-based scaffolds used by Curtin *et al*. and Duarte Campos *et al*. in their respective studies^51,52^. The stiffness properties of the printed hydrogels without bioinks, determined by Young’s modulus as previously described, increased in line with AlgMA concentrations^43^, and no significant differences were found comparing the Young’s modulus values of each stiffness at 37 °C or at room temperature, which confirms that all measurements carried out in human tumors, mice tumors and hydrogels are comparable at room temperature. However, the Young’s modulus decrease in composite hydrogels bioprinted and incubated with SK-N-BE(2) for 24 days with 1% and 2% AlgMA indicates an increased elasticity in these hydrogels^53^. Among other factors, this could be linked to cell-mediated hydrogel degradation as cells grow, releasing matrix metalloproteinases (MMP)^54^, but also to cell proliferation and migration through the hydrogel, and the softer consistency of the tumor cells compared to the ECM^55,56^. Furthermore, elasticity is increased in tumors derived from NB cells inoculated orthotopically into mice, highlighting the differences between human tumors and animal tissues. Murine tumor models can simulate certain physiopathological conditions but do not completely resemble human disease, owing to differences in tumor microenvironment, ECM composition and high cell density^57^. Despite that, the similarities found between the elastic properties of 1% AlgMA hydrogels and those present in HR-NB and xenograft NB models prove an advantage for prospective 3D bioprinted models. Moreover, since the fabricated composite hydrogels show viscoelastic behavior in the range of the stiffness described in neurons, normal epithelial colonies and adipocytes (from 0.1 to 1 kPa)^58^ and have adjustable stiffness properties, they provide promising insights for future design of fine-tuning mimetic NB 3D models. As expected, porosity in the bioprinted hydrogels decreased alongside increased alginate concentration, as hydrogels became stiffer and more compact^43^. Although final porosity varied only minimally between stiffness conditions, it increased particularly with culturing time in the stiffest bioprinted hydrogels. Porosity could be generated by cell activity, driving hydrogel degradation with MMP^54^ or generating gaps by cluster detachment. Moreover, porosity could be a consequence of the sample preanalytical processing (fixation, paraffin embedding and sectioning). However, further studies are needed to confirm whether MMPs are responsible for the strong porosity observed and to pinpoint the causes of highest susceptibility in the stiffest hydrogels.

In accordance with current knowledge of biology and cell cultures, we expected that cell cluster density and occupancy would increase over time in our 3D models, due to cell migration and proliferation^52^. Moreover, given that ECM with high stiffness has been associated with aggressiveness in NB^38^, we predicted cell cluster density and occupancy would increase with stiffness. We found growth in cell cluster occupancy, but density tended to decrease over time, meaning that cluster generation processes like cell migration and cluster division were underrepresented compared to cell cluster growth, death and detachment processes. Additionally, contrary to expectation, we observed that cell cluster density and occupancy were reduced with increasing stiffness^1,7^. Compared to the permissive characteristics of the low stiffness hydrogels, high stiffness equals a restrictive ECM, which apparently hinders initial cell migration and growth. In restricted environments cell migration is affected not only by matrix stiffness but also by initial matrix pore size, as compared to the cell nucleus size^59^, and by pore shape^60^. Despite this, we report very small pore size in our models, almost without change between different stiffness levels, which could inhibit cell spreading^15^. In fact, cell nucleus morphology changes were also minimal, suggesting that in our models, cell migration would not be driven by nucleus deformation related to matrix pore size, although these pores could facilitate matrix tunneling by cell MMP activity^54,61^. Nevertheless, temporal cell growth increase in the stiffest hydrogels equilibrates the occupancy values between the different modelled conditions. Observed growth capacity can be explained by cell adaptation, but also by a loss of stiffness to a more permissive state, related to cell-mediated hydrogel degradation. Persistent reduction in small cluster density in the highest stiffness levels at 4 weeks suggests that malignant neuroblasts would be preparing genetic and epigenetic pathways to promote cell migration.

One explanatory cellular behavior pattern could be permissive cell growth at lowest stiffness with later cell death in unadapted cells, with highest stiffness conversely only allowing cell proliferation in cells capable of adapting. Alternatively, cellular loss at lowest stiffness could also be explained by cellular detachment, as these hydrogels are less compact scaffolds. Initial culture times and lowest stiffness conditions are the most variable ones, suggesting that cells have an initial adaptive process while they are proliferating, migrating and dying until reaching a stable condition. Another observation is that as the most physiologically similar bioprinted hydrogel, 1% AlgMA condition shows lower cluster density variation and reaches final density values quickly.

We also noted greater cell proliferation as stiffness and culture time increased, regardless of the size of clusters analyzed, which as expected indicates that no cell contact inhibition of proliferation mechanisms are related to the stiffness of the scaffolding^62^. In fact, we observed that a similar increase of cells in the proliferative phase and with high mRNA processing index occurs at 4 weeks culture, nearly reaching the expression values found in 2D cell cultures. The high MKI values obtained are consistent with the characteristic SK-N-BE(2) aggressiveness found in malignant NB^29–31^, and also demonstrate that this cell line could be adequately replicated in a high stiffness ECM proliferative tumor model. The higher increase found in the antiapoptosis marker (Bcl2), cell proliferation and mRNA processing index, compared with those found in the apoptosis marker (Bax) and the individual increases in stiffness, confirm that the cells adapt more efficiently with the highest stiffness. Interestingly, Bcl2 and Bax expression patterns in 3D structures replicated human tumor behavior more accurately than 2D cultured cells, in which both biomarkers were overexpressed.

Having characterized the cellular dynamics of SK-N-BE(2) cell line according to time and stiffness in a 3D *in vitro* NB model, we successfully generated long-term 3D culture platforms for an aggressive NB cell line and established preliminary mechanical conditions physiologically similar to NB tumors in the 3D model. We conclude that increased stiffness hinders initial cell proliferation and loses part of its impact over time by cell-mediated hydrogel degradation and cell growth recovery. Including Schwann cells and other components of the cellular matrix like immune cells and vessels in the bioinks could improve the physiopathological preliminary 3D *in vitro* model of aggressive NB, adding ECM components to better mimic tumor mechanotransduction. All these systems will enhance the use of 3D models in biomedicine, providing useful insights for tailoring biomimetic systems to each physiological and/or tumor tissue, and can be used to advance preclinical research.

## Supplementary information

Supplementary information.

## Data Availability

The datasets generated and/or analyzed during the current study are available from the corresponding author on reasonable request.
