# Supplementary material for: A three-dimensional bioprinted model to evaluate the effect of stiffness on neuroblastoma cell cluster dynamics and behavior
Source: Sci Rep. 2020 Apr 14;10:6370. doi: 10.1038/s41598-020-62986-w (PMC7156444; doi:10.1038/s41598-020-62986-w)
Supplement: Supplementary file 1 — Supplementary information. [file 41598_2020_62986_MOESM1_ESM.pdf]

Title: A three-dimensional bioprinted model to evaluate the effect of stiffness on neuroblastoma cell cluster dynamics and behavior

Authors: Ezequiel Monferrer[a, b]#, Susana Martín-Vañó[a, b]#, Aitor Carretero[a]#, Andrea García-Lizarribar[c, e], Rebeca Burgos-Panadero[a, b], Samuel Navarro[a, b], Josep Samitier[c, d, e]*, Rosa Noguera[a, b]*

Supplementary Figure legends

Supplementary Figure 1. Variation in percentage of the Young's modulus at 37ºC from the Young's Modulus calculated at room temperature in the three hydrogel types. b) Characterization of the Young's modulus of the composite hydrogels, cohort of HR-NB and orthotopic NB mice tumors. Comparative Young´s modulus of 5% gelatin methacrylated with 0%, 1% and 2% methacrylated alginate in absence and presence of incubated SK-N-BE(2) cells. Measurements of each hydrogel were performed in quintuplicate. Values are plotted as mean and standard deviation. Statistical analysis using *t-student* test: ^{ns} *p*-value > 0.05, ** *p*-value < 0.01, ****p*-value < 0.001, comparing SK-NB-E (2) and no cell-hydrogels, $^{\#\#\#}$*p*-value < 0.001, comparing NB patients vs SK-NB-E(2) hydrogels and $^{\$\$\$}$*p*-value < 0.001, comparing orthotopic NB mice tumors vs SK-NB-E(2) hydrogels.

Supplementary Figure 2. Example of hydrogel cluster measurement using Pannoramic Viewer. Red circle marks each hydrogel cluster. Hydrogel HE staining (15x).

Supplementary Figure 3. Diagram of methodology used. 1. Hydrogel bioprinting; 2. Young's modulus measurement and paraffin embedding; 3. Immunohistochemistry; 4. Digitation and microscopic studies; 5. Analysis and quantification.

Supplementary Figure 4. Characterization of bioprinted hydrogels. Percentage of clusters (a1-a5), cluster density (b1-b5) and percentage of occupancy (c1-c5) for each cluster size (ranges: <400, 400-2.000, > 2,000 μm^2). (a, b, c: 1-3) Study over time within the same stiffness; (a, b, c: 4-5) Study of stiffness over the same timeframe. (d1-d2) Porosity of bioprinted hydrogels: d1) Study over time within the same stiffness; d2) Study of stiffness within the same timeframe. Values are plotted as mean and standard error of the mean.

Supplementary Figure 5. Study of cell proliferation in each cluster size (ranges: <400, 400-2.000, > 2,000 μm^2). Percentage of negative and positive cells for a) Ki67 and b) PTBP1. Results

expressed as percentage cells represent total number of cells, differentiating between cluster size. White bars: negative cells; Black bars: positive cells. Statistical analysis using X^2 test: * *p*-value < 0.05, ** *p*-value < 0.01, ****p*-value < 0.001.

Supplementary Figures

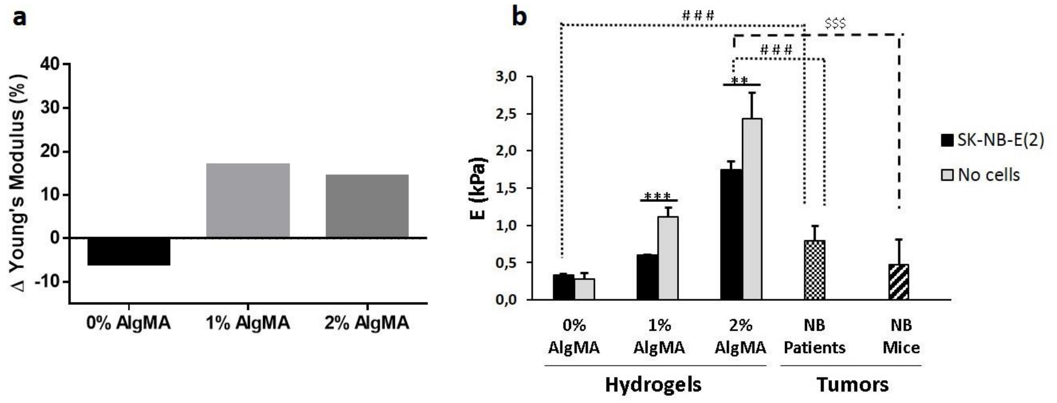

Supplementary Figure 1

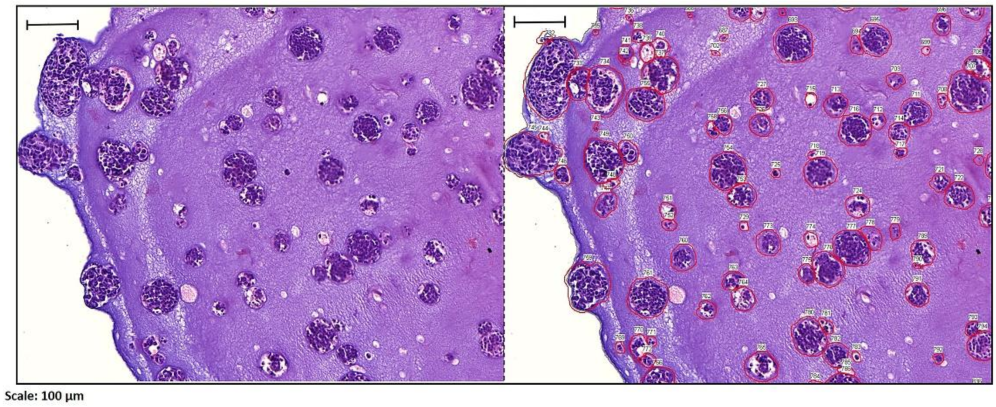

Supplementary Figure 2

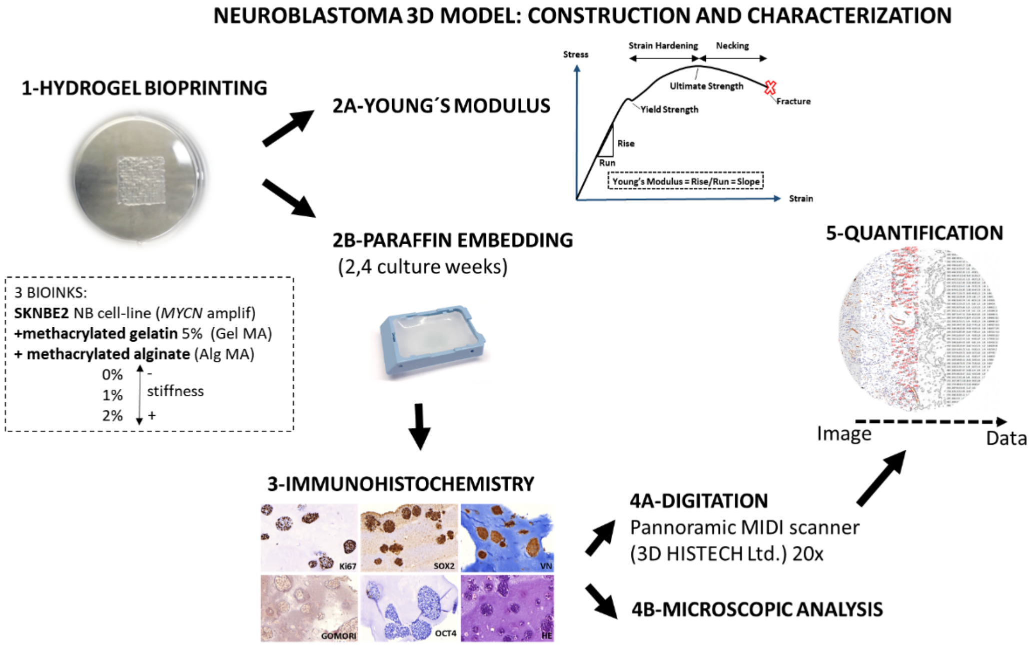

Supplementary Figure 3

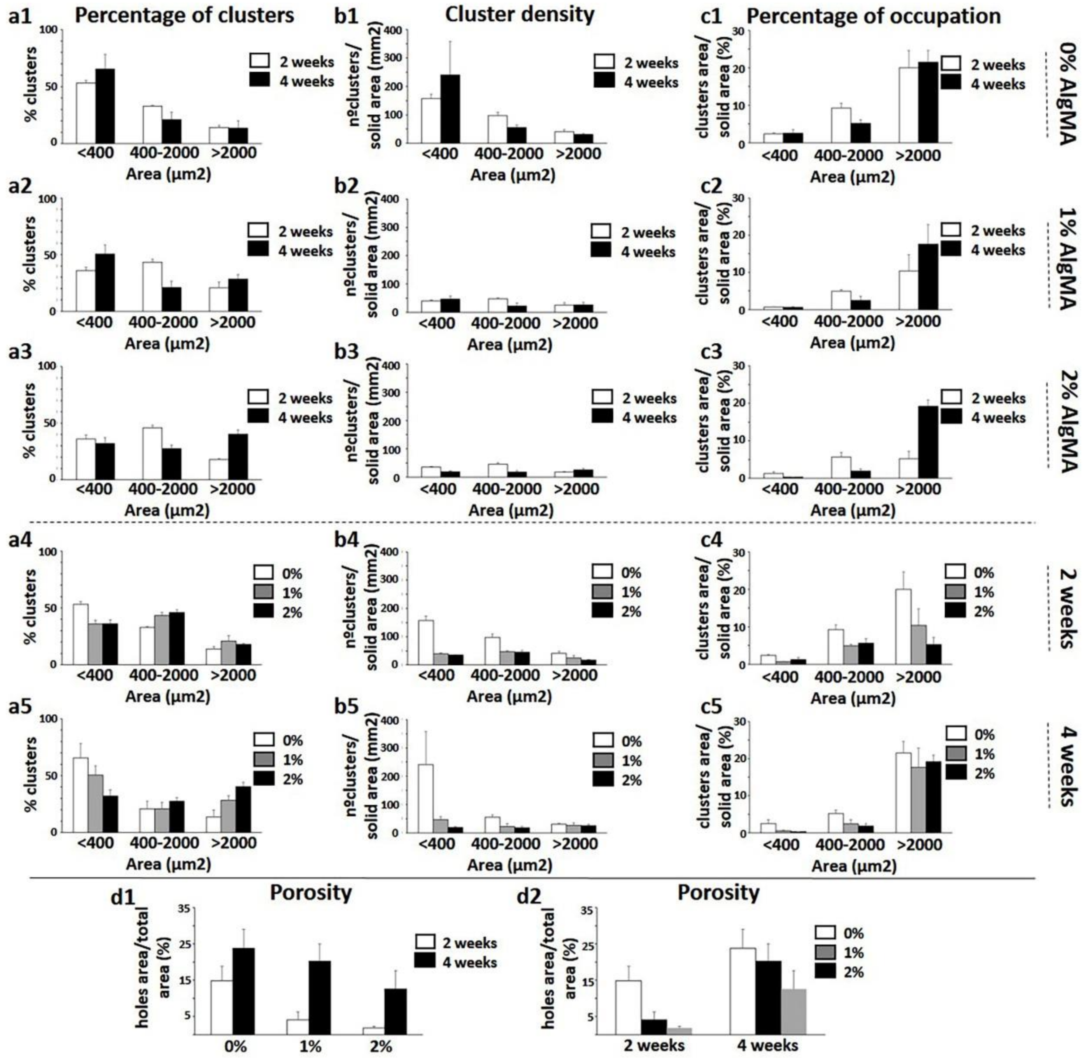

Supplementary Figure 4

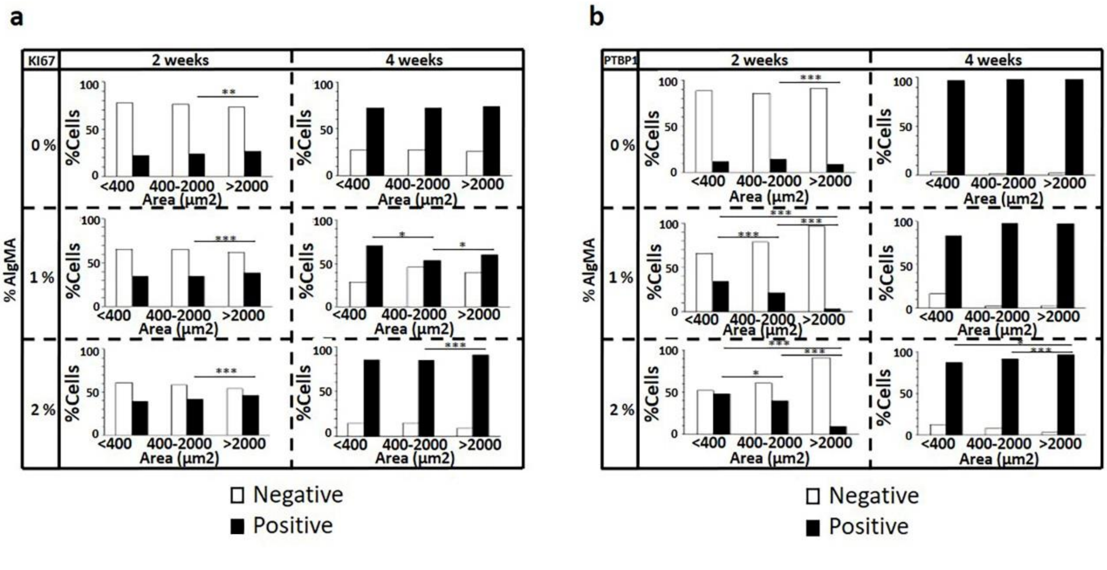

Supplementary Figure 5
